# Associations between cardiometabolic multimorbidity and cerebrospinal fluid biomarkers of Alzheimer’s disease pathology in cognitively intact adults: the CABLE study

**DOI:** 10.1186/s13195-024-01396-w

**Published:** 2024-02-06

**Authors:** Qiong-Yao Li, He-Ying Hu, Gao-Wen Zhang, Hao Hu, Ya-Nan Ou, Liang-Yu Huang, An-Yi Wang, Pei-Yang Gao, Li-Yun Ma, Lan Tan, Jin-Tai Yu

**Affiliations:** 1 Department of Neurology, Qingdao Municipal Hospital, Qingdao University, No.5 Donghai Middle Road, Qingdao, China; 2 Department of Thoracic Surgery, The Fourth Affiliated Hospital of China Medical University, Shenyang, China; 3 Department of Neurology and National Center for Neurological Disorders, Huashan Hospital, State Key Laboratory of Medical Neurobiology and MOE Frontiers Center for Brain Science, Shanghai Medical College, Fudan University, No. 12 Wulumuqi Road, Shanghai, China

**Keywords:** Alzheimer’s disease, Biomarkers, Cerebrospinal fluid, Cardiometabolic multimorbidity, Tau, Phosphorylated tau

## Abstract

**Background:**

Cardiometabolic multimorbidity is associated with an increased risk of dementia, but the pathogenic mechanisms linking them remain largely undefined. We aimed to assess the associations of cardiometabolic multimorbidity with cerebrospinal fluid (CSF) biomarkers of Alzheimer’s disease (AD) pathology to enhance our understanding of the underlying mechanisms linking cardiometabolic multimorbidity and AD.

**Methods:**

This study included 1464 cognitively intact participants from the Chinese Alzheimer’s Biomarker and LifestylE (CABLE) database. Cardiometabolic diseases (CMD) are a group of interrelated disorders such as hypertension, diabetes, heart diseases (HD), and stroke. Based on the CMD status, participants were categorized as CMD-free, single CMD, or CMD multimorbidity. CMD multimorbidity is defined as the coexistence of two or more CMDs. The associations of cardiometabolic multimorbidity and CSF biomarkers were examined using multivariable linear regression models with demographic characteristics, the *APOE ε4* allele, and lifestyle factors as covariates. Subgroup analyses stratified by age, sex, and *APOE ε4* status were also performed.

**Results:**

A total of 1464 individuals (mean age, 61.80 years; age range, 40–89 years) were included. The markers of phosphorylated tau-related processes (CSF P-tau181: *β* = 0.165, *P* = 0.037) and neuronal injury (CSF T-tau: *β* = 0.065, *P* = 0.033) were significantly increased in subjects with CMD multimorbidity (versus CMD-free), but not in those with single CMD. The association between CMD multimorbidity with CSF T-tau levels remained significant after controlling for Aβ42 levels. Additionally, significantly elevated tau-related biomarkers were observed in patients with specific CMD combinations (i.e., hypertension and diabetes, hypertension and HD), especially in long disease courses.

**Conclusions:**

The presence of cardiometabolic multimorbidity was associated with tau phosphorylation and neuronal injury in cognitively normal populations. CMD multimorbidity might be a potential independent target to alleviate tau-related pathologies that can cause cognitive impairment.

**Supplementary Information:**

The online version contains supplementary material available at 10.1186/s13195-024-01396-w.

## Background

Cardiometabolic multimorbidity is defined as the coexistence of two or more cardiometabolic diseases (CMDs), including hypertension, diabetes, heart disease (HD), and stroke [1]. These diseases share several risk factors, and one may influence another bidirectionally across the life course [2–4]. Currently, patients rarely have one disease in isolation, but instead tend to have multiple coexisting conditions that develop concurrently [5]. CMD multimorbidity is one of the most common multimorbidity patterns in the middle-aged and elderly population, causing a higher symptom burden and worse subsequent outcomes [5, 6]. It is generally accepted that CMDs are independent contributors to poor cognition and dementia [7–10]. Recent studies have shown that patients with CMD multimorbidity have a faster cognitive decline and a higher risk of dementia than patients with single CMD [2, 11]. However, little is known about the role of CMD multimorbidity in the pathogenesis of cognitive deterioration. Alzheimer’s disease (AD), the most common cause of dementia, is characterized pathologically by extracellular neuritic plaques and intracellular neurofibrillary tangles [12]. The progression of neurofibrillary tangle pathology and the loss of synapses have been shown to be closely associated with the severity of cognitive impairment in AD patients [13]. These changes begin decades before the earliest clinical symptoms emerge [14]. Cerebrospinal fluid (CSF) biomarkers can reflect these AD pathologies in the brain and offer the promise of identifying preclinical AD in vivo in cognitively intact individuals [15].

Over the last few decades, a large number of previous studies focused on the relationships between various CMDs and CSF biomarkers of AD pathology. Two studies have reported positive associations between hypertension with amyloid plaques and neurofibrillary tangles [16, 17]. Diabetes and AD have been reported to share some common pathophysiological features, such as β-amyloid (Aβ) deposition and elevated levels of phosphorylated tau [18]. Persistent atrial fibrillation and heart failure might lead to reduced left ventricular ejection fraction, which was shown to be associated with greater tau phosphorylation and neurodegeneration in cognitively normal adults [19–23]. Reduced Aβ42 and increased tau-related biomarkers, which are pathological hallmarks of AD, were also observed in patients with acute ischemic stroke [24]. What the above studies did not show, however, is whether their complex interactions influence the relationships between themselves and AD pathologies. Subsequently, we compared the changes in CSF biomarkers (Aβ42, phosphorylated tau [P-tau181], total tau [T-tau]) between participants with single CMD and those with CMD multimorbidity, with CMD-free participants as the reference. And we hypothesized that CMD multimorbidity rather than single CMDs was associated with lower levels of CSF Aβ42 and higher levels of CSF tau-related biomarkers in cognitively intact adults.

Therefore, in this study, we divided participants into CMD-free, single CMD, and CMD multimorbidity groups based on CMD status and then performed (1) the multivariable linear regression (MLR) models to investigate the associations of single CMDs and CMD multimorbidity with CSF AD biomarkers in cognitively intact participants; (2) stratified analyses by age, sex, and *APOE ɛ4* status; and (3) sensitivity analyses to determine whether the conclusions were robust and which specific CMD combinations had associations with CSF AD biomarkers.

## Methods

### Participants

All data for this study were derived from the Chinese Alzheimer’s Biomarker and LifestylE (CABLE) study, an ongoing large and independent cohort initiated in 2017 [25]. The study focused on the risk factors and biomarkers of AD in the Chinese Han population. The Han Chinese participants aged 40–90 years were enrolled from Qingdao Municipal Hospital, Shandong Province, China. The participants of this study were drawn from clinical samples, recruited through direct communication from the inpatient wards. The main exclusionary criteria were central nervous system infection, epilepsy, head trauma, psychological disorders, malignant tumors, and family history of genetic diseases. Demographic information, clinical history, and laboratory findings were extracted from the included individuals’ electronic medical records (EMR) at the time of admission, and each participant underwent a structured interview and structured questionnaire data collection at enrolment. CSF and blood samples were collected the next day after an overnight fast following collection of clinical findings/history data. The CABLE study complies with the Declaration of Helsinki, and the Institutional Ethics Committee of Qingdao Municipal Hospital approved the protocol. Written informed consent was obtained from all the participants.

Participants were screened for mild cognitive impairment using China Modified Mini-Mental State Examination (CM-MMSE: score of ≤ 24 for secondary school or above, ≤ 20 for less than elementary school or elementary school completed, ≤ 17 for no formal schooling) [26]. In this study, we excluded patients with cognitive impairment. We also excluded patients with a diagnosis of dementia or a history of dementia based on EMR. CMD multimorbidity has been found in previous studies to potentially increase the risk of depression, and depression has also been reported to be associated with AD pathology [27, 28]. Thus, we used the Hamilton Rating Scale for Depression to assess depression (score > 7) and excluded patients with depression to control for the potential confounding effect of depression on our study findings. The CABLE study had a total of 2047 cognitively intact adults without significant symptoms of depression, of which 583 participants who did not provide CSF biomarker data or whose data exceeded four times the standard deviation were excluded. Ultimately, our study included 1464 individuals.

### Clinical evaluation and covariates

CMDs were identified at baseline using multiple sources of evidence. Hypertension was ascertained on the basis of self-reported hypertension history, use of antihypertensive medications, and diagnoses from EMR. Diabetes was identified based on self-reported diabetes medication use, diagnoses from EMR, clinical history of diabetes or fasting glucose ≥ 7.0 mmol/L. History of HD was defined as atrial fibrillation, heart failure, coronary artery disease, or ischemic heart disease based on self-reported history of a previous physician diagnosis and diagnoses from EMR. And stroke was defined as intracerebral hemorrhage or cerebral infarction based on self-reported medical history and clinical diagnoses extracted from the EMR. See Supplementary Table 1 for more details. Based on CMD status, participants were divided into 3 categories: CMD-free, single CMD, and CMD multimorbidity (≥ 2 CMDs). We included further information on the duration of CMD for each participant as part of this ancillary study. The data presented in this study regarding the duration of CMD was collected through self-report questionnaires and by reviewing past medical history from EMR. And we categorized disease length into two groups: a short disease course, defined as less than 5 years of disease, and a long disease course, which exceeded 5 years.

Basic information included age (continuous), sex (female versus male), years of education (continuous), MMSE score (continuous), apolipoprotein E allele (*APOE*) -*ε4* (carriers versus non-carriers) status, body mass index (BMI: continuous), cigarette use (non-smoker versus current/former smoker), alcohol use (no/occasional drinking versus drinking), and physical activity (active versus inactive). Alcohol consumption was divided into regular drinking (≥ 1 time/week), occasional drinking (< 1 time/week), and never drinking. Physical activity was divided into active physical activity (≥ 1 time/week) and inactive physical activity (< 1 time/week). The QIAamp® DNA Blood Mini Kit (250) was used to extract DNA from peripheral blood cells. DNA samples were stored at − 80 °C until assay. *APOE ε4* status was determined by genotyping rs7412 and rs429358 using restriction fragment-length polymorphisms analysis. *APOE ε4* carriers were referred to the individuals with at least one *ε4* allele. BMI was measured using standard methods. Cigarette use, alcohol use, and physical activity were ascertained by a self-report questionnaire.

### CSF AD biomarker measurements

Lumbar punctures were performed by a qualified physician to obtain CSF samples. CSF samples were processed within 2 h of the collection. After being centrifuged at 2000 × *g* for 10 min, they were aliquoted and then stored at − 80 °C, avoiding repeated freeze-thawing. CSF Aβ42, Aβ40, P-tau181, and T-tau levels were determined with the ELISA kits (Innotest β-AMYLOID (1–42) [catalog number: 81583]; β-AMYLOID (1–40) [catalog number: 81585]; PHOSPHO-TAU (181p) [catalog number: 81581]; hTAU-Ag [catalog number: 81579]; Fujirebio, Ghent, Belgium). Absorbance measurements were taken at 450 nm with the Multiskan FC Microplate Photometer (Thermo Scientific) and analyzed with SkanIt Software 6.1 RE for Microplate Readers RE, ver. 6.1.0.51. The optical density of the standard curve was plotted and protein concentration was calculated with a 4PL curve analysis. For Aβ42, P-tau181, and T-tau, undiluted CSF was used. For Aβ40, it was diluted 100-fold. The lowest limit of detection (LLOD) values for Aβ42, Aβ40, P-tau181, and T-tau were found to be 62.5 pg/mL, 6.4 pg/mL, 15.6 pg/mL, and 38.3 pg/mL, respectively. The highest limited of detection (HLOD) values for Aβ42, Aβ40, P-tau181, and T-tau were found to be 4599.0 pg/mL, 1000.0 pg/mL, 1060.0 pg/mL, and 2620.0 pg/mL, respectively. All assays were performed in duplicate by experienced operators blinded to clinical information. The within-batch coefficients of variations were < 5%. The inter-batch coefficients of variations were < 20%.

### Statistical analysis

Outliers (outside four standard deviations) were removed. A total of 21 individuals identified as outliers were removed from further analyses. Differences between groups were compared by the Kruskal–Wallis test or analysis of variance for continuous variables and the chi-square test for categorical variables. The Box-Cox transformation and the *z* score standardization for CSF AD biomarkers were performed. Group differences of CSF biomarkers were identified by ANOVA and pairwise comparisons were conducted using the Tukey HSD post hoc test.

MLR analyses were performed to observe the association between the CMD counts with CSF AD biomarkers after adjusting for covariates. With the CMD-free group as the reference, the associations of single CMD and CMD multimorbidity with CSF AD biomarkers were also further verified in MLR models. Furthermore, the associations of individual CMDs (hypertension, diabetes, HD, stroke) with CSF biomarkers were also separately explored in MLR models. All the above regression analyses utilized the MLR models adjusting for age, sex, education years, MMSE, *APOE ɛ4* status, BMI, cigarette use, alcohol use, and physical activity. In our study, we examined effect modification by age, sex, and *APOE ε4* status by creating an interaction term between the exposure and each stratification variable. If the *P*-value of the interaction term was below 0.10, effect modification was considered. We also conducted subgroup analyses stratified by age (mid-life and late-life, < 65 and ≥ 65 years), sex (female and male), and *APOE ɛ4* status (non-carriers and carriers). Additionally, sensitivity analyses were performed by (1) analyzing the associations between various CMD combinations with CSF AD biomarkers, (2) additionally controlling for CSF Aβ42 to examine whether the results were robust, and (3) observing the effect of disease length on the associations of CMD status and CSF AD biomarkers.

*R* (version 4.0.5) and *R* package (e.g., “car”, “stats”, “ggplot2”, “pheatmap”, “mice”) were used for data analysis and visualization. A two-tailed *P* value < 0.05 was considered statistically significant, except for tests of interaction, where *P* value < 0.10 was considered significant. The missing covariates were imputed using the “mice” package in R, employing multiple imputation [29].

## Results

### Characteristics of the study population

**Fig. 1:**
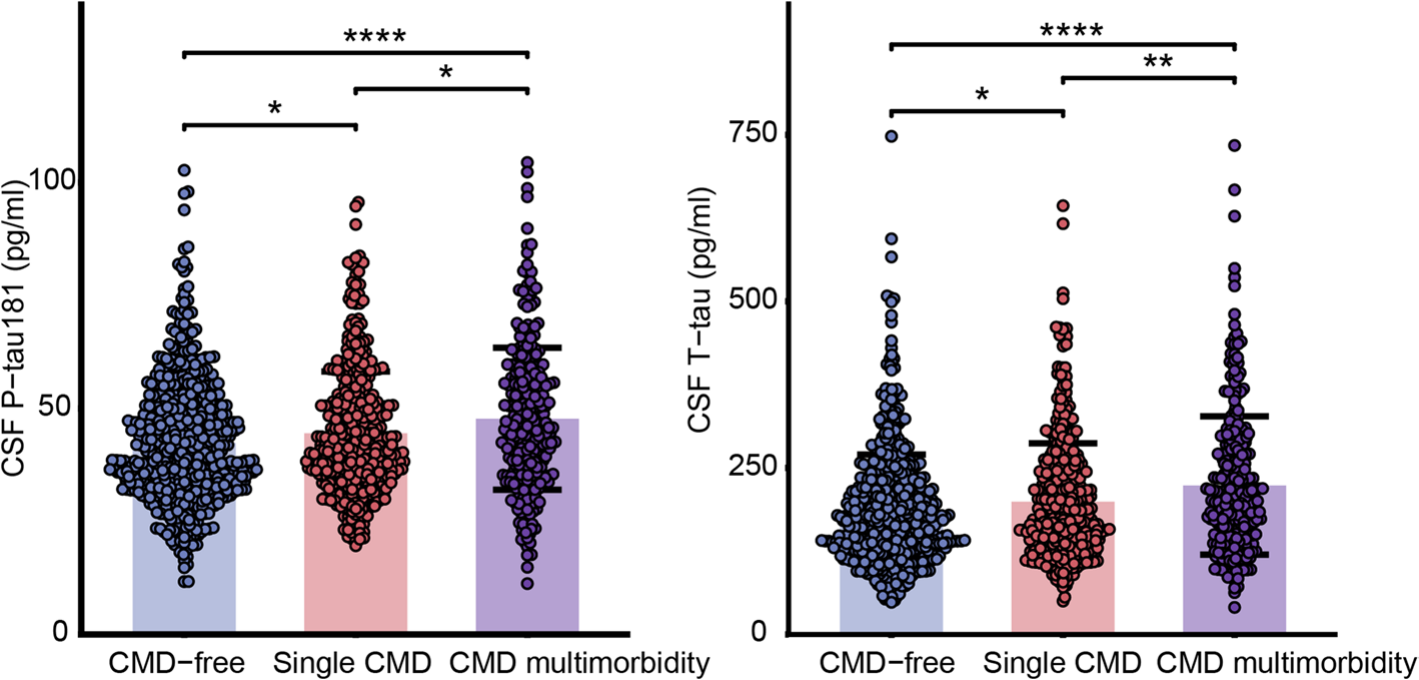
CSF tau-related biomarkers in three CMD status groups. Bar graphs show mean, and error bars represent standard deviations. Bee swarm plots visualize the distribution of biomarkers data. CSF tau-related biomarkers were Box-Cox power transformed, and then, the *z*-score normalized. One-way ANOVA was used to test group differences in biomarkers, followed by the Tukey HSD posthoc test. CSF P-tau181, T-tau significantly differed among groups (all *P* values < 0.05). We observed that the levels of CSF P-tau181 and T-tau were highest in the CMD multimorbidity group, followed by an intermediate level in the single CMD group, and lowest in the CMD-free group (all *P* values < 0.05). *P* > 0.05: NS; *P* ≤ 0.05: *; *P* ≤ 0.01: **; *P* ≤ 0.001: ***; *P* ≤ 0.0001: ****. Abbreviations: CMD, cardiometabolic disease; CSF, cerebrospinal fluid

We demonstrate the demographic, clinical, lifestyle, and biomarker characteristics in Table 1. Of the total 1464 participants, 310 (21.17%) individuals had CMD multimorbidity, 454 (31.01%) had single CMD, and 700 (47.81%) were CMD-free/healthy controls. Compared to CMD-free/healthy participants, those with CMD multimorbidity were more likely to be older and have higher BMI and lower MMSE scores. No differences among the three groups were found in terms of sex, education years, alcohol use, physical activity, or *APOE ε4* allele carrier status. Concentrations of CSF P-tau181 and T-tau in individuals with CMD multimorbidity were higher than those in CMD-free/healthy participants and participants with single CMD (Fig. 1).

**Table 1:** Characteristics of the study population by cardiometabolic disease status (*n* = 1464)

	Total (*n* = 1464)	CMD-free (*n* = 700)	Single CMD (*n* = 454)	CMD multimorbidity (*n* = 310)	*P*-value
Age, years	61.80 ± 10.16	58.27 ± 9.98	63.42 ± 9.17	67.40 ± 8.75	**< 0.001**^**a**^
Female	629 (42.96%)	287 (41.00%)	207 (45.59%)	135 (43.55%)	0.297^b^
Education, years	9.49 ± 4.35	9.65 ± 4.25	9.09 ± 4.42	9.70 ± 4.41	0.063^a^
BMI, kg/m^2^	25.62 ± 3.75	24.76 ± 3.72	26.20 ± 3.46	26.70 ± 3.79	**< 0.001**^**a**^
Cigarette use, yes	399 (27.30%)	209 (29.94%)	104 (22.96%)	86 (27.83%)	**0.033**^**b**^
Alcohol use, yes	416 (28.50%)	206 (29.47%)	131 (28.92%)	79 (25.57%)	0.434^b^
Physically active	811 (55.70%)	392 (56.40%)	242 (53.54%)	177 (57.28%)	0.520^b^
*APOE* ε4 carriers	191 (15.00%)	82 (13.80%)	62 (15.62%)	47 (16.73%)	0.487^b^
MMSE	27.75 ± 2.25	27.94 ± 2.19	27.64 ± 2.38	27.51 ± 2.15	**0.008**^**a**^
CSF AD biomarkers					
Aβ42, pg/ml	341.53 ± 212.79	336.90 ± 211.92	341.28 ± 213.52	352.36 ± 213.97	0.533^c^
P-tau181, pg/ml	44.15 ± 14.12	42.35 ± 13.32	44.49 ± 13.70	47.70 ± 15.71	**< 0.001**^**c**^
T-tau, pg/ml	198.54 ± 90.32	186.98 ± 83.14	199.24 ± 87.70	223.60 ± 103.81	**< 0.001**^**c**^
Aβ42/ Aβ40	0.06 ± 0.05	0.06 ± 0.05	0.06 ± 0.04	0.05 ± 0.03	0.210^c^
P-tau181/ Aβ42	0.19 ± 0.17	0.19 ± 0.15	0.20 ± 0.17	0.21 ± 0.20	0.376^c^
T-tau/ Aβ42	0.87 ± 0.88	0.81 ± 0.71	0.89 ± 1.01	0.98 ± 1.02	0.0579^c^

Data are presented as means ± standard deviations or number (proportion %)

*Abbreviations*: *BMI* Body mass index, *APOE ε4* Apolipoprotein E ε4 allele

^a^The difference among the groups was examined by the Kruskal–Wallis test

^b^The difference among the groups was examined by the chi-square test

^c^The difference among the groups was examined by the analysis of variance (raw data were Box-Cox-transformed). Proportions of missing data: BMI (1.71%), education attainment (0.27%), *APOE ε4* (13.11%), cigarette use (0.27%), alcohol use (0.20%), and physical activity (0.55%)

### The relationships of CMD multimorbidity with CSF biomarkers

Among the cognitively intact participants, the greater the number of CMD disease, the higher the levels of CSF P-tau181 and T-tau (P-tau181: *β* = 0.078, *P* = 0.044; T-tau: *β* = 0.030, *P* = 0.047, Table 2). When grouping participants according to CMD status, we found that the levels of CSF tau-related biomarkers (P-tau181: *β* = 0.165, *P* = 0.037; T-tau: *β* = 0.065, *P* = 0.033, Table 2) were significantly higher in patients with CMD multimorbidity than those in CMD-free participants, independent of age, sex, years of education, MMSE, *APOE ε4* carrier status, BMI, cigarette use, alcohol use, and physical activity. And we found no significant associations between single CMDs and CSF AD biomarkers (Supplementary Table 2).

**Table 2:** Associations of cardiometabolic disease status and CSF AD biomarkers in cognitively intact participants

Variable	Aβ42		P-tau181		T-tau		Aβ42/Aβ40		P-tau181 /Aβ42		T-tau/Aβ42	
	*β*	*P* value	*β*	*P* value	*β*	*P* value	*β*	*P* value	*β*	*P* value	*β*	*P* value
Number of CMD (continuous)	0.070	0.504	**0.078**	**0.044**	**0.030**	**0.047**	−0.007	0.637	0.007	0.879	0.012	0.661
**CMD status**												
CMD-free (*n* = 700)	Ref.		Ref.		Ref.		Ref.		Ref.		Ref.	
Single CMD (*n* = 454)	−0.036	0.840	0.047	0.475	0.009	0.721	−0.008	0.751	0.049	0.528	0.020	0.670
CMD multimorbidity (*n* = 310)	0.170	0.428	**0.165**	**0.037**	**0.065**	**0.033**	−0.014	0.650	0.002	0.981	0.022	0.696

Adjusted for age, sex, education years, MMSE, *APOE ε4* carrier status, BMI, cigarette use, alcohol use, and physical activity. The statistically significant results were bolded

*Abbreviations*: *CMD* Cardiometabolic disease, *MMSE* Mini-Mental State Examination, *APOE* Apolipoprotein E, *BMI* Body mass index

### Interactions and stratified analyses by age, sex, and *APOE ε4* carrier status

**Fig. 2:**
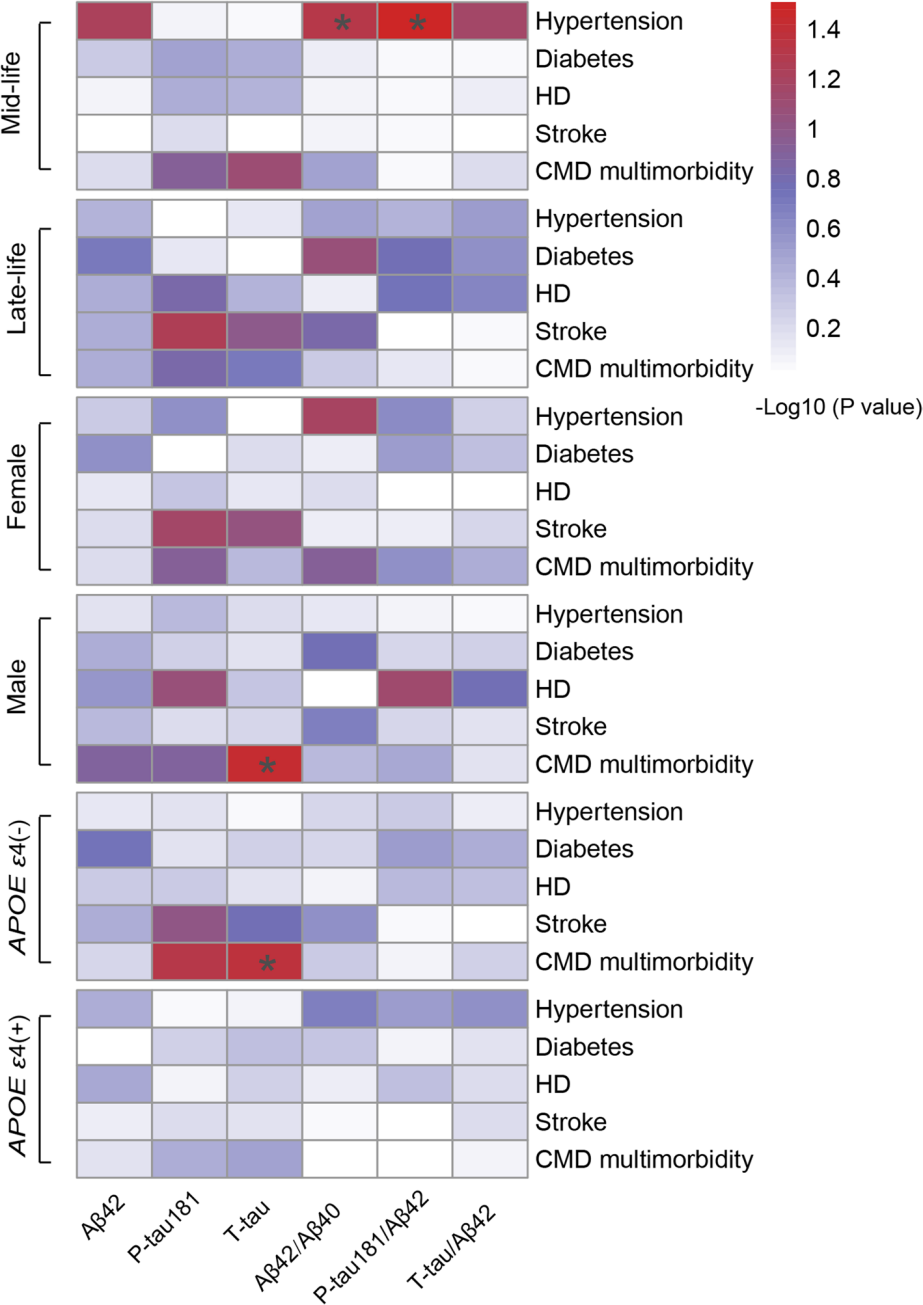
Stratified analyses of associations between CSF AD biomarkers and CMD status. Multiple linear regression models were employed with adjustment for age, sex, education years, MMSE, *APOE ε4* carrier status, BMI, cigarette use, alcohol use, and physical activity. In stratified analyses, CMD multimorbidity showed significant or suggestive associations with CSF T-tau levels in males, and *APOE ε4* non-carriers. The association between hypertension and CSF AD biomarkers was found in mid-life, but not in late-life. Abbreviations: CMD, cardiometabolic disease; CSF, cerebrospinal fluid; AD, Alzheimer’s disease; MMSE, Mini-Mental State Examination; *APOE ε4*, apolipoprotein E; BMI, body mass index; HD, heart disease

The interaction analysis expressed that the association between CMD multimorbidity and P-tau181/Aβ42 ratio was affected by sex, while the associations between hypertension and CSF AD biomarkers were affected by age and sex (Supplementary Table 3). The distribution of patients in different subgroups according to CMD status is shown in Supplementary Fig. 1. The proportion of participants with CMD multimorbidity was higher in the late-life group (33.17%) than in the mid-life group (12.56%), but the proportion showed no significant differences across sex and *APOE ε4* carrier status (21.46% in female vs. 20.96% in male; 20.62% in *APOE ε4* non-carriers vs. 24.34% in *APOE ε4* carriers). Results revealed that these associations between CMD multimorbidity with CSF T-tau levels were found in male and *APOE ε4* non-carrier groups (Fig. 2). In addition, we only found the associations between hypertension with a lower Aβ42/40 ratio and a higher P-tau181/Aβ42 ratio in midlife, but not in late life. Details are available in Supplementary Table 4.

### Sensitivity analyses

Our results showed that two CMD combinations (i.e., hypertension and diabetes, hypertension and HD) had associations with CSF P-tau181 levels. We did not conduct multivariable regression analyses for some CMD combinations due to their small sample sizes (Supplementary Table 5). Subsequently, given the strong association of amyloid pathology with tau pathology, we performed sensitivity analyses by additionally controlling for CSF Aβ42 levels [30]. We found that the association of CMD multimorbidity with CSF T-tau levels (T-tau: *β* = 0.059, *P* = 0.045) remained significant, but the association with CSF P-tau181 levels became nonsignificant (P-tau181: *β* = 0.151, *P* > 0.050, Supplementary Table 6).

We also examined the impact of disease duration on the relationship between CMD status and CSF AD biomarkers. In the group with a short disease course, we did not identify any significant associations between the presence of CMD events and CSF AD biomarkers. However, in the group with a long disease course, our findings revealed several associations between specific CMD conditions and CSF AD biomarkers. Specifically, diabetes was found to be associated with higher levels of CSF T-tau. HD was associated with a higher P-tau181/Aβ42 ratio. Two CMD combinations, namely hypertension and diabetes, and hypertension and HD, were associated with higher levels of CSF P-tau181. The CMD combination of hypertension and diabetes was also linked to higher CSF T-tau levels and a lower Aβ42/Aβ40 ratio. The combination of hypertension and stroke was associated with higher levels of CSF Aβ42. Lastly, the CMD combination of hypertension, HD, and stroke was associated with higher CSF T-tau levels. Details are available in Supplementary Table 7.

## Discussion

In this sample of cognitively intact individuals, we investigated the cross-sectional associations of CMD multimorbidity with CSF AD biomarkers (including Aβ42, P-tau181, T-tau, Aβ42/Aβ40, P-tau181/Aβ42, T-tau/Aβ42). We found that CMD multimorbidity was associated with higher levels of CSF tau-related biomarkers. The association of CMD multimorbidity and CSF T-tau levels remained significant after controlling for Aβ42 levels. Moreover, patients with specific combinations of CMD, such as hypertension and diabetes or hypertension and heart disease, showed significantly elevated levels of tau-related biomarkers, particularly if they had a longer disease course. Our results indicated that in cognitively intact participants, CMD multimorbidity could be closely related to tau phosphorylation and neuronal injury, which are characteristic features of AD pathology.

Our findings are in agreement with the previous studies suggesting that cardiovascular disease or multimorbidity was associated with biomarkers of neurodegeneration or/and pathologic tau [31–34]. In the population-based Mayo Clinic Study of Aging, a stronger association was found between multimorbidity with neuronal damage in cognitively intact participants [31]. Subsequently, the authors also defined biomarker combinations (A-N-, A+N-, A-N+, A+N+) to assess the association between multimorbidity with preclinical Alzheimer’s disease stages and suspected non-amyloid pathophysiology, yielding the result that multimorbidity was associated with neurodegeneration [32]. A study on adults with subjective cognitive decline from the INSIGHT-preAD cohort reported that multimorbidity was associated with lower hippocampal volumes and lower brain metabolism [33]. Furthermore, metabolic multimorbidity patterns are thought to be closely related to tau pathology [35].

Although AD is characterized by both amyloid and tau pathologies, it has been shown that tau pathology is more strongly associated with cognitive decline than amyloid pathology [36, 37]. In the current study, CMD multimorbidity could confer an increased risk of dementia through its association with tau phosphorylation. CSF P-tau181 is recognized as a specific biomarker for AD [38]. The amyloid cascade hypothesis is the leading model for AD pathogenesis, which proposed that amyloid deposition in the brain is the initiating factor in AD pathogenesis, leading to subsequent neurofibrillary tangles, neuronal damage, and cognitive decline [39, 40]. The association between CMD multimorbidity and P-tau181 might represent the downstream impact of the association between CMD multimorbidity and Aβ42. Thus, our result could be explained by the low sensitivity of current CSF Aβ42 assays than the CSF P-tau181 and T-tau assays, or its inability to detect the earliest abnormal Aβ moieties [41]. CSF T-tau is considered a non-specific biomarker indicating neurodegeneration [38]. Several previous studies have suggested that the biomarkers of neurodegeneration may appear independently and ahead of amyloid pathology [42, 43]. Recent studies have indicated that a certain fraction of cognitively normal individuals with normal Aβ exhibits abnormal levels of biomarkers of neurodegeneration [44]. These findings support our result that the association between CMD multimorbidity and CSF T-tau levels is robust after correction for Aβ42.

Although we found CMD multimorbidity was associated with elevated CSF P-tau181 and T-tau levels, the precise mechanisms are not clear. Dynamic interactions among neurons, glial cells, and vascular cells contribute to the maintenance of the homeostasis of the brain’s microenvironment. CMD multimorbidity could influence this homeostasis and cause an imbalance of vascular regulatory mechanisms, changes in cerebral blood flow, and eventually cerebral hypoperfusion [45–48]. These changes could exacerbate tau pathology by damaging neurons, neuron-glial interactions, and the blood-brain barrier. Several reasons might account for the association between CMD multimorbidity and tau pathology. Firstly, the reduced cerebral blood flow caused by CMDs could promote neuronal damage, resulting in oxidative stress, mitochondrial dysfunction, and calcium deregulation, which may exacerbate tau pathology [49]. In response to the central nervous system damage, microglia may phagocytize extracellular tau but in parallel, release tau seeds, which might induce tau aggregation and promote its propagation [50]. Secondly, the reduction of the clearance of tau pathology was also a major factor that should not be ignored. On the one hand, oxidative stress could lead to or aggravate mitochondrial dysfunction and altered mitochondrial autophagy, which inhibits the clearance of tau aggregates [51, 52]. On the other hand, CMD multimorbidity may cause blood-brain barrier dysfunction which plays a key role in immune surveillance of the central nervous system and various pathological changes in the brain [53]. Moreover, the increased blood-brain barrier permeability may promote the entrance of neurotoxic substances into the brain tissues, which may exacerbate tau pathology [54]. Finally, CMDs might be not only a cause of tau-related pathologies but also a consequence. A previous study suggested that tau played a central role in stroke [55]. Therefore, there might be a vicious circle of CMD-tau interrelation.

Our stratified analyses showed stronger associations of CMD multimorbidity with CSF T-tau levels in male, and *APOE ε4* non-carrier subgroups. There were no significant differences in the prevalence of CMD multimorbidity across sex and *APOE ɛ4* status. Our stronger association in men was consistent with two previous studies showing a stronger association of multimorbidity with mild cognitive impairment and brain hypometabolism in men [56, 57]. A possible explanation for these differences could be related to variations in brain structure and function between genders. Previous research has demonstrated that older males have a higher frequency of whole brain and frontal lobe atrophy compared to females [58]. It is important to note that sex differences have also been reported for the age-dependent decline in the volume of cortical as well as subcortical gray matter, with faster progression in males [59]. A second possible explanation is that more severe work stress, independent of the lifestyle risk factors, might explain the higher brain pathology among males with CMD multimorbidity [60]. Our stronger effect for *APOE ɛ4* non-carriers might be partially due to some mechanistic commonalities shared by *APOE ɛ4* effects and CMD multimorbidity. A second possible explanation is that the sequencing of biomarkers of core AD pathology in the wider population may be different from that in *APOE ɛ4*-positive subjects. Early tau pathology may be more prevalent in *APOE ɛ4* non-carriers, whereas early amyloid pathology is more prevalent in *APOE ɛ4* carriers [41]. Moreover, it is worth noting that our study had a limited number of *APOE ε4* carriers, with only 191 individuals (15%). This limitation may increase the risk of false-negative results. Furthermore, we identified significant differences in the Aβ42/Aβ40 ratio and P-tau181/Aβ42 ratio between individuals with and without hypertension in mid-life. Reduced Aβ42/Aβ40 ratio and elevated P-tau181/Aβ42 ratio are suggestive of underlying Aβ pathology [61]. There is accumulating evidence indicating that mid-life hypertension is associated with an increased risk of AD, which may promote the progression of AD pathology [62]. It has been suggested that hypertension interacts with amyloid pathways, interfering with amyloid clearance through dysfunction of the blood-brain barrier and increasing amyloid deposition and plaques via elevated amyloidogenic processing of the amyloid precursor protein through β-secretase [63]. However, in late life, mild hypertension may have a protective effect against AD-associated neuropathologic lesions. This effect may be attributed to the natural decrease in the elasticity of the blood vessel wall with age, where mild hypertension could potentially help maintain cerebral blood flow [64]. These results were not found in previous studies on hypertension and AD pathology, possibly because the presence of co-morbidities was overlooked in the studies and the co-morbid population was mistakenly studied as a hypertensive population.

The aggregate effect of multiple co-occurring CMDs in brain pathology may differ from the simple summation of their individual effects. In our study, the association of hypertension with amyloid pathology was only found in mid-life patients with hypertension. However, after considering disease length, we found that the CMD combination of hypertension and diabetes was linked to a lower Aβ42/Aβ40 ratio, while the combination of hypertension and stroke was associated with higher levels of CSF Aβ42. It seems unlikely that CMD multimorbidity is protective against amyloid positivity. One plausible explanation is that the complex relationship between CMDs is likely to have weakened this association. Furthermore, significantly elevated tau-related biomarkers were only observed in patients with two CMD combinations (i.e., hypertension and diabetes, hypertension and HD). We found these two groups constituted more than half of all patients with CMD multimorbidity, while other CMD multimorbidity groups were underrepresented. This disparity in sample size could potentially affect the statistical power and reliability of the findings regarding the impact of the various CMDs or CMD combinations on brain pathology. However, at the same time, this implies that other CMDs or CMD combinations may not be as prevalent in the general population as hypertension and these two CMD combinations. This phenomenon could be also observed in previous studies [65, 66]. The finding underlines the importance of clustering disease factors. In addition, the duration of the disease is likely an important factor. Our findings suggest that the presence of CMD conditions may have varying effects on CSF AD biomarkers depending on the duration of the disease. This result, however, should be interpreted with caution given the possible recall bias regarding CMD duration.

Our study has several strengths. This is a large-scale study to explore the associations between CMD multimorbidity and CSF AD biomarkers. And we also adjusted for demographic characteristics, the *APOE* ε4 allele, and lifestyle factors, which were previously shown to be associated with cognitive function. In addition, given that Aβ pathology is often thought to influence the propagation of tau pathology, we also corrected for CSF Aβ42 levels and found that the association between CMD multimorbidity and CSF T-tau levels was robust. This study also has several limitations. Firstly, the causalities between CMD multimorbidity and CSF AD biomarkers cannot be figured out in this study due to the cross-sectional design. Secondly, since all the included participants in this study were Han Chinese, caution is needed in generalizing these findings to other ethnic groups. In addition, we had to admit that selection bias may be present in the study cohort since recruitment was performed only in one hospital. To provide more reliable and generalized information, we recognize the need to collaborate with other centers in future studies. By adopting a multicenter approach with a larger sample size, we can mitigate selection bias and provide a more comprehensive understanding of the topic. Finally, the limited number of participants with CMDs other than hypertension may have restricted the analyses, and future studies with a larger sample size should be conducted to confirm our findings.

## Conclusions

In conclusion, CMD multimorbidity was associated with higher levels of tau-related biomarkers. The association between CMD multimorbidity with CSF T-tau levels remained significant after controlling for Aβ42 levels. These findings support our hypothesis that CMD multimorbidity was closely related to a higher tau burden. To be more specific, two CMD combinations (i.e., hypertension and diabetes, hypertension and HD) were found to be associated with CSF P-tau181 levels. CMD multimorbidity (especially the two combinations: hypertension and diabetes, hypertension and HD) may be potential targets for the prevention of tau pathology.

### Supplementary Information

**Additional file 1: Supplementary Table 1.** Diagnosis of cardiometabolic diseases. **Supplementary Table 2.** Associations of single cardiometabolic disease and CSF AD biomarkers in cognitively intact participants. **Supplementary Table 3.** Interaction effects analyses of associations between CMD status with CSF AD biomarkers. **Supplementary Table 4.** Subgroup analyses of associations between CMD status with CSF AD biomarkers. **Supplementary Table 5.** Associations between the varying CMDs combinations with CSF P-tau181 and T-tau. **Supplementary Table 6.** Sensitivity analyses of associations between CMD status with CSF tau-related biomarkers additionally adjusting for Aβ42. **Supplementary Table 7.** Sensitivity analyses of associations between CMD status grouped by the length of disease with CSF AD biomarkers. **Supplementary Figure 1.** The distribution of patients in different subgroups according to CMD status.

## Data Availability

The datasets used during the current study are available from the corresponding author upon reasonable request.

## Abbreviations

CSF: Cerebrospinal fluid
AD: Alzheimer’s disease
CABLE: Chinese Alzheimer’s Biomarker and LifestylE
CMD: Cardiometabolic diseases
HD: Heart diseases
Aβ: β-Amyloid
P-tau: Phosphorylated tau
T-tau: Total tau
MLR: Multiple linear regression
EMR: Electronic medical records
CM-MMSE: China Modified Mini-Mental State Examination
BMI: Body mass index
*APOE*: Apolipoprotein E allele
LLOD: Lowest limit of detection
HLOD: Highest limited of detection
