# Supplementary material for: Associations between cardiometabolic multimorbidity and cerebrospinal fluid biomarkers of Alzheimer’s disease pathology in cognitively intact adults: the CABLE study
Source: Alzheimers Res Ther. 2024 Feb 6;16:28. doi: 10.1186/s13195-024-01396-w (PMC10848421; doi:10.1186/s13195-024-01396-w)
Supplement: Supplementary file 1 — Additional file 1: Supplementary Table 1. Diagnosis of cardiometabolic diseases. Supplementary Table 2. Associations of single cardiometabolic disease and CSF AD biomarkers in cognitively intact participants. Supplementary Table 3. Interaction effects analyses of associations between CMD status with CSF AD biomarkers. Supplementary Table 4. Subgroup analyses of associations between CMD status with CSF AD biomarkers. Supplementary Table 5. Associations between the varying CMDs combinations with CSF P-tau181 and T-tau. Supplementary Table 6. Sensitivity analyses of associations between CMD status with CSF tau-related biomarkers additionally adjusting for Aβ42. Supplementary Table 7. Sensitivity analyses of associations between CMD status grouped by the length of disease with CSF AD biomarkers. Supplementary Figure 1. The distribution of patients in different subgroups according to CMD status. [file 13195_2024_1396_MOESM1_ESM.docx]

**Supplementary Materials**

**Supplementary Table 1. Diagnosis of cardiometabolic diseases**

**Supplementary Table 2. Associations of single cardiometabolic disease and CSF AD biomarkers in cognitively intact participants**

**Supplementary Table 3. Interaction effects analyses of associations between CMD status with CSF AD biomarkers**

**Supplementary Table 4.** **Subgroup analyses of associations between CMD status with CSF AD biomarkers**

**Supplementary Table 5. Associations between the varying CMDs combinations with CSF P-tau181 and T-tau**

**Supplementary Table 6. Sensitivity analyses of associations between CMD status with CSF tau-related biomarkers additionally adjusting for Aβ42**

**Supplementary Table 7. Sensitivity analyses of associations between CMD status grouped by the length of disease with CSF AD biomarkers**

**Supplementary Figure 1. The distribution of patients in different subgroups according to CMD status**

**Supplementary Table 1. Diagnosis of cardiometabolic diseases**

| CMD | Diagnostic criteria |
| --- | --- |
| **Hypertension** | Self-reported medical history, use of antihypertensive medications, clinical diagnoses extracted from the EMR, SBP ≥ 140 or DBP ≥ 90 mmHg |
| **Diabetes** | Self-reported medical history, use of glucose-lowering medications, clinical diagnoses extracted from the EMR, laboratory data (fasting glucose ≥7.0 mmol/L) |
| **HD**   - Atrial fibrillation - Heart failure - Ischemic heart disease | Self-reported medical history, clinical diagnoses extracted from the EMR |
| **Stroke**   - Ischemic stroke - Haemorrhagic stroke | Self-reported medical history, clinical diagnoses extracted from the EMR |

Abbreviations: CMD, cardiometabolic disease; EMR, electronic medical record; HD, heart disease

**Supplementary Table 2.** **Associations of single cardiometabolicdisease and CSF AD biomarkers in cognitively intact participants**

|  | Aβ42 | | P-tau181 | | T-tau | | Aβ42/40 | | P-tau181/ Aβ42 | | T-tau/ Aβ42 | |
| --- | --- | --- | --- | --- | --- | --- | --- | --- | --- | --- | --- | --- |
|  | β | *P* | β | *P* | β | *P* | β | *P* | β | *P* | β | *P* |
| CMD-free (n=700) | Ref. |  | Ref. |  | Ref. |  | Ref. |  | Ref. |  | Ref. |  |
| Hypertension (n=278) | -0.157 | 0.457 | 0.023 | 0.772 | -0.005 | 0.866 | -0.029 | 0.334 | 0.092 | 0.314 | 0.037 | 0.512 |
| Diabetes (n=103) | 0.402 | 0.186 | 0.064 | 0.565 | 0.033 | 0.445 | 0.037 | 0.395 | -0.123 | 0.35 | -0.06 | 0.459 |
| HD (n=65) | -0.336 | 0.371 | 0.074 | 0.593 | 0.007 | 0.89 | -0.012 | 0.827 | 0.163 | 0.317 | 0.081 | 0.417 |
| Stroke (n=8) | 0.876 | 0.389 | 0.514 | 0.171 | 0.206 | 0.153 | 0.175 | 0.234 | -0.097 | 0.826 | 0.003 | 0.991 |

Multiple linear regression models were conducted with all models adjusted for age, sex, education, Mini-Mental State Examination (MMSE), apolipoprotein E (*APOE ε4*) carrier status, body mass index (BMI), cigarette use, alcohol use, and physical activity.

**Supplementary Table 3. Interaction effects analyses of associations between CMD status with CSF AD biomarkers**

|  | Aβ42 | | P-tau181 | | T-tau | | Aβ42/40 | | P-tau181/ Aβ42 | | T-tau/ Aβ42 | |
| --- | --- | --- | --- | --- | --- | --- | --- | --- | --- | --- | --- | --- |
| interaction | β | *P* | β | *P* | β | *P* | β | *P* | β | *P* | β | *P* |
| Age x |  |  |  |  |  |  |  |  |  |  |  |  |
| Hypertension | 0.604 | 0.151 | -0.064 | 0.686 | -0.031 | 0.606 | **0.113** | **0.064** | **-0.326** | **0.074** | **-0.197** | **0.079** |
| Diabetes | 0.213 | 0.734 | -0.220 | 0.346 | -0.066 | 0.465 | 0.130 | 0.150 | -0.215 | 0.427 | -0.109 | 0.514 |
| HD | -0.702 | 0.351 | 0.337 | 0.231 | 0.078 | 0.475 | -0.044 | 0.686 | 0.407 | 0.211 | 0.239 | 0.235 |
| Stroke | 0.710 | 0.762 | 1.046 | 0.234 | 0.185 | 0.585 | 0.308 | 0.365 | 0.177 | 0.861 | 0.036 | 0.954 |
| CMD multimorbidity | -0.164 | 0.695 | -0.067 | 0.666 | -0.050 | 0.411 | 0.059 | 0.326 | 0.021 | 0.907 | -0.012 | 0.915 |
| Sex x |  |  |  |  |  |  |  |  |  |  |  |  |
| Hypertension | 0.025 | 0.951 | **-0.282** | **0.061** | -0.049 | 0.396 | 0.076 | 0.201 | -0.133 | 0.456 | -0.055 | 0.613 |
| Diabetes | -0.216 | 0.730 | 0.009 | 0.970 | -0.047 | 0.597 | 0.084 | 0.349 | 0.107 | 0.693 | 0.013 | 0.938 |
| HD | -0.469 | 0.527 | 0.413 | 0.129 | 0.038 | 0.720 | 0.025 | 0.815 | 0.433 | 0.177 | 0.165 | 0.404 |
| Stroke | -0.065 | 0.978 | -1.302 | 0.130 | -0.490 | 0.140 | 0.106 | 0.754 | -0.523 | 0.606 | -0.476 | 0.445 |
| CMD multimorbidity | 0.510 | 0.196 | -0.130 | 0.372 | -0.004 | 0.939 | 0.081 | 0.156 | **-0.289** | **0.090** | -0.136 | 0.196 |
| *APOE* *ε4* x |  |  |  |  |  |  |  |  |  |  |  |  |
| Hypertension | -0.909 | 0.176 | -0.070 | 0.779 | 0.013 | 0.888 | -0.149 | 0.100 | 0.334 | 0.224 | 0.249 | 0.139 |
| Diabetes | -0.509 | 0.538 | 0.072 | 0.814 | 0.038 | 0.750 | 0.018 | 0.875 | 0.231 | 0.526 | 0.167 | 0.466 |
| HD | -1.039 | 0.336 | -0.017 | 0.966 | -0.066 | 0.694 | -0.104 | 0.498 | 0.397 | 0.398 | 0.182 | 0.540 |
| Stroke | -1.461 | 0.619 | -1.036 | 0.367 | -0.005 | 0.991 | -0.246 | 0.574 | 0.133 | 0.917 | 0.353 | 0.652 |
| CMD multimorbidity | -0.226 | 0.695 | 0.018 | 0.939 | 0.007 | 0.939 | -0.045 | 0.571 | 0.088 | 0.705 | 0.055 | 0.702 |

Multiple linear regression models were conducted with all models adjusted for age, sex, education, Mini-Mental State Examination (MMSE), apolipoprotein E (*APOE* *ε4*) carrier status, body mass index (BMI), cigarette use, alcohol use, and physical activity. The statistically significant results were bolded.

**Supplementary Table 4. Subgroup analyses of associations between CMD status with CSF AD biomarkers**

|  | Aβ42 | | P-tau181 | | T-tau | | Aβ42/40 | | P-tau181/ Aβ42 | | T-tau/ Aβ42 | |
| --- | --- | --- | --- | --- | --- | --- | --- | --- | --- | --- | --- | --- |
| Subgroups | β | *P* | β | *P* | β | *P* | β | *P* | β | *P* | β | *P* |
| Mid-life |  |  |  |  |  |  |  |  |  |  |  |  |
| Hypertension | -0.510 | 0.058 | 0.021 | 0.833 | -0.006 | 0.878 | **-0.078** | **0.048** | **0.253** | **0.031** | 0.130 | 0.071 |
| Diabetes | 0.244 | 0.516 | 0.137 | 0.321 | 0.048 | 0.363 | -0.015 | 0.788 | -0.016 | 0.921 | -0.010 | 0.921 |
| HD | -0.116 | 0.833 | -0.188 | 0.352 | -0.065 | 0.400 | 0.017 | 0.833 | -0.037 | 0.877 | -0.041 | 0.782 |
| Stroke | -0.041 | 0.983 | -0.357 | 0.624 | 0.012 | 0.965 | -0.054 | 0.853 | -0.116 | 0.893 | 0.017 | 0.975 |
| CMD multimorbidity | 0.160 | 0.613 | 0.181 | 0.119 | 0.078 | 0.081 | -0.047 | 0.317 | 0.017 | 0.903 | 0.038 | 0.655 |
| Late-life |  |  |  |  |  |  |  |  |  |  |  |  |
| Hypertension | 0.290 | 0.399 | -0.001 | 0.994 | -0.017 | 0.728 | 0.049 | 0.318 | -0.13 | 0.379 | -0.094 | 0.299 |
| Diabetes | 0.656 | 0.200 | -0.061 | 0.750 | -0.002 | 0.983 | 0.126 | 0.083 | -0.302 | 0.168 | -0.151 | 0.261 |
| HD | -0.475 | 0.366 | 0.281 | 0.150 | 0.065 | 0.393 | -0.018 | 0.808 | 0.300 | 0.183 | 0.169 | 0.223 |
| Stroke | 1.088 | 0.371 | 0.864 | 0.056 | 0.282 | 0.106 | 0.250 | 0.149 | -0.026 | 0.960 | 0.035 | 0.913 |
| CMD multimorbidity | 0.273 | 0.372 | 0.165 | 0.145 | 0.057 | 0.198 | 0.028 | 0.516 | -0.049 | 0.709 | -0.013 | 0.867 |
| Female |  |  |  |  |  |  |  |  |  |  |  |  |
| Hypertension | -0.215 | 0.501 | 0.135 | 0.260 | -0.003 | 0.951 | -0.085 | 0.066 | 0.163 | 0.238 | 0.053 | 0.534 |
| Diabetes | 0.572 | 0.259 | 0.005 | 0.981 | 0.036 | 0.627 | -0.020 | 0.783 | -0.230 | 0.292 | -0.103 | 0.451 |
| HD | -0.200 | 0.708 | -0.139 | 0.487 | -0.026 | 0.737 | -0.039 | 0.616 | -0.014 | 0.951 | 0.011 | 0.940 |
| Stroke | 0.933 | 0.649 | 1.389 | 0.071 | 0.505 | 0.088 | 0.093 | 0.754 | 0.232 | 0.793 | 0.285 | 0.604 |
| CMD multimorbidity | -0.162 | 0.629 | 0.196 | 0.119 | 0.038 | 0.424 | -0.076 | 0.116 | 0.161 | 0.263 | 0.082 | 0.362 |
| Male |  |  |  |  |  |  |  |  |  |  |  |  |
| Hypertension | -0.114 | 0.688 | -0.083 | 0.422 | -0.018 | 0.648 | 0.015 | 0.724 | 0.027 | 0.829 | 0.011 | 0.882 |
| Diabetes | 0.350 | 0.359 | 0.080 | 0.563 | 0.021 | 0.695 | 0.075 | 0.174 | -0.090 | 0.587 | -0.057 | 0.569 |
| HD | -0.592 | 0.271 | 0.335 | 0.086 | 0.052 | 0.490 | 0.001 | 0.991 | 0.414 | 0.076 | 0.195 | 0.170 |
| Stroke | 0.940 | 0.422 | 0.203 | 0.634 | 0.088 | 0.590 | 0.214 | 0.208 | -0.259 | 0.610 | -0.133 | 0.666 |
| CMD multimorbidity | 0.426 | 0.133 | 0.154 | 0.133 | **0.082** | **0.038** | 0.034 | 0.410 | -0.118 | 0.336 | -0.029 | 0.702 |
| *APOEɛ4* non-carriers |  |  |  |  |  |  |  |  |  |  |  |  |
| Hypertension | -0.083 | 0.720 | 0.034 | 0.694 | -0.005 | 0.892 | -0.017 | 0.602 | 0.067 | 0.502 | 0.017 | 0.780 |
| Diabetes | 0.456 | 0.176 | 0.055 | 0.660 | 0.028 | 0.564 | 0.027 | 0.577 | -0.150 | 0.305 | -0.080 | 0.376 |
| HD | -0.263 | 0.521 | 0.099 | 0.519 | 0.024 | 0.690 | -0.013 | 0.830 | 0.147 | 0.406 | 0.081 | 0.459 |
| Stroke | 0.990 | 0.375 | 0.688 | 0.096 | 0.215 | 0.174 | 0.185 | 0.250 | -0.066 | 0.892 | -0.017 | 0.954 |
| CMD multimorbidity | 0.130 | 0.586 | 0.180 | 0.050 | **0.070** | **0.042** | -0.022 | 0.517 | 0.025 | 0.810 | 0.037 | 0.552 |
| *APOEɛ4* carriers |  |  |  |  |  |  |  |  |  |  |  |  |
| Hypertension | -0.580 | 0.365 | 0.039 | 0.876 | 0.021 | 0.814 | -0.109 | 0.205 | 0.275 | 0.299 | 0.183 | 0.266 |
| Diabetes | 0.039 | 0.959 | 0.150 | 0.567 | 0.085 | 0.436 | 0.077 | 0.467 | 0.058 | 0.860 | 0.092 | 0.663 |
| HD | -0.962 | 0.332 | -0.088 | 0.810 | -0.094 | 0.543 | -0.035 | 0.801 | 0.326 | 0.448 | 0.129 | 0.636 |
| Stroke | -0.667 | 0.803 | -0.470 | 0.634 | 0.164 | 0.664 | -0.047 | 0.906 | 0.093 | 0.936 | 0.337 | 0.641 |
| CMD multimorbidity | 0.258 | 0.661 | 0.221 | 0.357 | 0.085 | 0.323 | 0.007 | 0.933 | 0.001 | 0.995 | 0.028 | 0.854 |

Multiple linear regression models were conducted with all models adjusted for age, sex, education, Mini-Mental State Examination (MMSE), apolipoprotein E (*APOE* *ε4*) carrier status, body mass index (BMI), cigarette use, alcohol use, and physical activity. The statistically significant results were bolded.

**Supplementary Table 5. Associations between the varying** **CMDs combinations with CSF P-tau and T-tau**

|  |  | P-tau181 | |  | T-tau | |
| --- | --- | --- | --- | --- | --- | --- |
|  |  | β | *P* value |  | β | *P* value |
| CMD-free (n=700) |  | Ref. |  |  | Ref. |  |
| Hypertension + Diabetes (n=93) |  | **0.242** | **0.049** |  | **0.097** | **0.040** |
| Hypertension + HD (n=88) |  | **0.275** | **0.033** |  | 0.075 | 0.131 |
| Diabetes + HD (n=24) |  | 0.289 | 0.199 |  | 0.090 | 0.298 |
| Hypertension + Stroke (n=17) |  | -0.065 | 0.805 |  | 0.088 | 0.383 |
| Diabetes + Stroke (n=2) |  | - | - |  | - | - |
| HD + Stroke (n=2) |  | - | - |  | - | - |
| Hypertension + Diabetes + HD (n=46) |  | 0.109 | 0.518 |  | 0.061 | 0.343 |
| Hypertension + HD + Stroke (n=18) |  | 0.157 | 0.546 |  | 0.141 | 0.156 |
| Diabetes + HD + Stroke (n=2) |  | - | - |  | - | - |
| Hypertension + Diabetes + Stroke (n=4) |  | - | - |  | - | - |
| Hypertension + Diabetes + HD + Stroke (n=14) |  | -0.070 | 0.811 |  | -0.006 | 0.956 |

Multiple linear regression models were conducted with all models adjusted for age, sex, education, Mini-Mental State Examination (MMSE), apolipoprotein E (*APOE ε4*) carrier status, body mass index (BMI), cigarette use, alcohol use, and physical activity.

-:the number of events is <5, so the event rates are non-estimable

**Supplementary Table 6. Sensitivity analyses of associations between CMD status with CSF tau-related biomarkers additionally adjusting for Aβ42**

|  |  | P-tau181 | |  | T-tau | |
| --- | --- | --- | --- | --- | --- | --- |
|  |  | β | *P* value |  | β | *P* value |
| CMD-free (n=700) |  | Ref. |  |  | Ref. |  |
| Single CMD (n=454) |  | 0.050 | 0.435 |  | 0.010 | 0.679 |
| CMD multimorbidity (n=310) |  | 0.151 | 0.0503 |  | **0.059** | **0.045** |

Multiple linear regression models were conducted with all models adjusted for age, sex, education, Mini-Mental State Examination (MMSE), apolipoprotein E (*APOE* *ε4*) carrier status, body mass index (BMI), cigarette use, alcohol use, physical activity, and CSF Aβ42 levels

**Supplementary Table 7.** **Sensitivity analyses of associations between CMD status grouped by the length of disease with CSF AD biomarkers**

|  | | Aβ42 | | P-tau181 | | T-tau | | Aβ42/40 | | P-tau181/ Aβ42 | | T-tau/ Aβ42 | | |
| --- | --- | --- | --- | --- | --- | --- | --- | --- | --- | --- | --- | --- | --- | --- |
| Subgroups | | β | *P* | β | *P* | β | *P* | β | *P* | β | *P* | β | *P* |  |
| CMD-free (n=700) | Ref. | |  | Ref. |  | Ref. |  | Ref. |  | Ref. |  | Ref. |  |  |
|  |  | |  |  |  |  |  |  |  |  |  |  |  |  |
| **Short disease course (<5 y)** |  | |  |  |  |  |  |  |  |  |  |  |  |  |
| Hypertension (n=75) | -0.095 | | 0.780 | 0.118 | 0.144 | 0.038 | 0.463 | 0.003 | 0.960 | 0.157 | 0.343 | 0.070 | 0.493 |  |
| Diabetes (n=16) | -0.322 | | 0.642 | -0.257 | 0.120 | -0.113 | 0.285 | 0.133 | 0.209 | -0.073 | 0.830 | -0.041 | 0.846 |  |
| HD (n=20) | -0.358 | | 0.568 | -0.048 | 0.745 | -0.036 | 0.708 | -0.037 | 0.696 | 0.103 | 0.736 | 0.046 | 0.807 |  |
| Stroke (n=1) | - | | - | - | - | - | - | - | - | - | - | - | - |  |
| Hypertension + Diabetes (n=27) | 0.673 | | 0.215 | 0.137 | 0.290 | 0.086 | 0.302 | 0.030 | 0.720 | -0.223 | 0.399 | -0.099 | 0.546 |  |
| Hypertension + HD (n=16) | -0.071 | | 0.918 | 0.260 | 0.115 | 0.105 | 0.321 | -0.049 | 0.645 | 0.204 | 0.545 | 0.071 | 0.734 |  |
| Diabetes + HD (n=2) | - | | - | - | - | - | - | - | - | - | - | - | - |  |
| Hypertension + Stroke (n=2) | - | | - | - | - | - | - | - | - | - | - | - | - |  |
| Diabetes + Stroke (n=1) | - | | - | - | - | - | - | - | - | - | - | - | - |  |
| HD + Stroke (n=1) | - | | - | - | - | - | - | - | - | - | - | - | - |  |
| Hypertension + Diabetes + HD (n=2) | - | | - | - | - | - | - | - | - | - | - | - | - |  |
| Hypertension + HD + Stroke (n=5) | 0.381 | | 0.739 | 0.133 | 0.625 | 0.097 | 0.578 | -0.012 | 0.945 | -0.095 | 0.866 | -0.029 | 0.933 |  |
| Diabetes + HD + Stroke (n=0) | - | | - | - | - | - | - | - | - | - | - | - | - |  |
| Hypertension + Diabetes + Stroke (n=1) | - | | - | - | - | - | - | - | - | - | - | - | - |  |
| Hypertension + Diabetes + HD + Stroke (n=1) | - | | - | - | - | - | - | - | - | - | - | - | - |  |
|  |  | |  |  |  |  |  |  |  |  |  |  |  |  |
| **Long disease course (≥ 5 y)** |  | |  |  |  |  |  |  |  |  |  |  |  |  |
| Hypertension (n=153) | -0.025 | | 0.926 | 0.039 | 0.544 | -0.003 | 0.938 | -0.042 | 0.298 | 0.069 | 0.595 | 0.015 | 0.851 |  |
| Diabetes (n=26) | 1.050 | | 0.062 | 0.094 | 0.473 | **0.202** | **0.017** | 0.055 | 0.513 | -0.400 | 0.137 | -0.063 | 1.050 |  |
| HD (n=22) | -1.063 | | 0.081 | 0.167 | 0.241 | -0.005 | 0.954 | -0.099 | 0.273 | **0.642** | **0.027** | 0.300 | -1.063 |  |
| Stroke (n=0) | - | | - | - | - | - | - | - | - | - | - | - | - |  |
| Hypertension + Diabetes (n=49) | -0.349 | | 0.438 | **0.301** | **0.004** | **0.144** | **0.034** | **-0.157** | **0.019** | 0.389 | 0.071 | 0.232 | -0.349 |  |
| Hypertension + HD (n=58) | 0.030 | | 0.940 | **0.225** | **0.015** | 0.080 | 0.180 | 0.015 | 0.804 | 0.164 | 0.387 | 0.076 | 0.030 |  |
| Diabetes + HD (n=19) | 0.107 | | 0.871 | 0.097 | 0.527 | 0.037 | 0.707 | -0.033 | 0.738 | 0.011 | 0.972 | 0.008 | 0.107 |  |
| Hypertension + Stroke (n=14) | **1.457** | | **0.043** | 0.087 | 0.603 | 0.117 | 0.282 | 0.052 | 0.629 | -0.586 | 0.088 | -0.270 | 0.201 |  |
| Diabetes + Stroke (n=1) | - | | - | - | - | - | - | - | - | - | - | - | - |  |
| HD + Stroke (n=1) | - | | - | - | - | - | - | - | - | - | - | - | - |  |
| Hypertension + Diabetes + HD (n=32) | 0.635 | | 0.212 | 0.052 | 0.664 | 0.024 | 0.758 | 0.031 | 0.683 | -0.282 | 0.246 | -0.147 | 0.635 |  |
| Hypertension + HD + Stroke (n=11) | 1.131 | | 0.169 | 0.288 | 0.133 | **0.254** | **0.040** | 0.112 | 0.361 | -0.296 | 0.450 | -0.047 | 1.131 |  |
| Diabetes + HD + Stroke (n=0) | - | | - | - | - | - | - | - | - | - | - | - | - |  |
| Hypertension + Diabetes + Stroke (n=3) | - | | - | - | - | - | - | - | - | - | - | - | - |  |
| Hypertension + Diabetes + HD + Stroke (n=13) | 0.014 | | 0.987 | -0.079 | 0.676 | -0.016 | 0.893 | -0.001 | 0.993 | -0.055 | 0.887 | -0.001 | 0.014 |  |

Multiple linear regression models were conducted with all models adjusted for age, sex, education, Mini-Mental State Examination (MMSE), apolipoprotein E (*APOE ε4*) carrier status, body mass index (BMI), cigarette use, alcohol use, and physical activity.

-:the number of events is <5, so the event rates are non-estimable

**Supplementary Figure 1. The distribution of patients in different subgroups according to CMD status
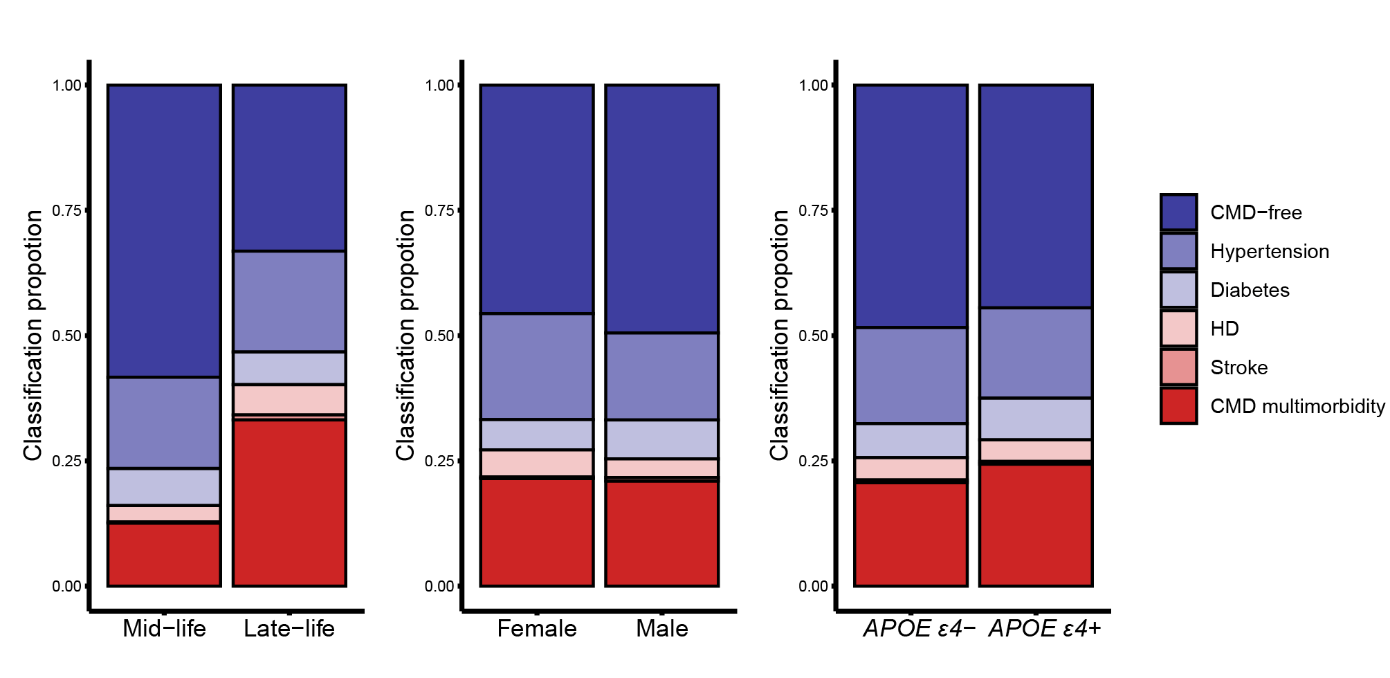
**

There was an increase in proportion of patients with CMD multimorbidity with age (12.56% in mid-life vs. 33.17% in late-life). There were no significant differences in population proportions by sex and in different *APOE ε4* subgroups (21.46% in female vs. 20.96% in male; 20.62% in *APOE ε4* non-carriers vs. 24.34% in *APOE ε4* carriers).
